# Neutralizing VHH Antibodies Targeting the Spike Protein of PEDV

**DOI:** 10.3390/vetsci11110533

**Published:** 2024-11-01

**Authors:** Li Zhang, Wei Miao, Mo Zhou, Miao Lin, Changyao Fu, Zhi Wu, Xinnuo Lei, Jialong Xu, Shinuo Cao, Shanyuan Zhu

**Affiliations:** 1 Jiangsu Key Laboratory for High-Tech Research and Development of Veterinary Biopharmaceuticals, Engineering Technology Research Center for Modern Animal Science and Novel Veterinary Pharmaceutic Development, Jiangsu Agri-Animal Husbandry Vocational College, Taizhou 225300, China; lzhang@jsahvc.edu.cn (L.Z.); mo_zhou@jsahvc.edu.cn (M.Z.); zhiwu@jsahvc.edu.cn (Z.W.); xlei@jsahvc.edu.cn (X.L.); 2 College of Veterinary Pharmacy, Jiangsu Agri-Animal Husbandry Vocational College, Taizhou 225300, China; 2021010402@jsahvc.edu.cn (W.M.); 202210677@jsahvc.edu.cn (M.L.); 2300790304@jsahvc.edu.cn (C.F.); 3 Medical School, Nanjing University, Nanjing 210093, China; dg1935007@smail.nju.edu.cn

**Keywords:** porcine epidemic diarrhea virus, spike (S) protein, neutralizing antibody, nanobody, VHH

## Abstract

Porcine epidemic diarrhea virus (PEDV) is a highly contagious coronavirus that infect pigs’ intestinal epithelial cells, causing high morbidity and mortality. Due to the rapid mutation of PEDV, vaccine efficacy is uncertain, prompting exploration of alternative treatments. Nanobodies, also known as variable heavy chain domains of heavy chain-only antibodies (VHHs), offer significant potential in biomedical applications due to their small size and high specificity. In this study, yeast two-hybrid technology was employed to screen for eight specific VHH sequences targeting the PEDV S protein from a synthetically constructed nanobody yeast library. The VHH genes were then cloned into expression plasmids for recombinant protein production, and the resulting VHHs (termed PEDV S-VHHs) were purified. Indirect immunofluorescence assay (IFA) and Western blotting analysis confirmed that these VHHs specifically bind to both PEDV and its S protein. Neutralization assays demonstrated that seven PEDV S-VHHs exhibited potent neutralizing activity against PEDV. Additionally, a combination of these seven antibodies showed enhanced antiviral effects. Preliminary predictions were also made regarding the binding sites between these VHHs and PEDV. The PEDV S-VHHs described in this study hold potential as candidates for the prevention and treatment of PEDV infection.

## Simple Summary

This study presents a novel method for screening nanobodies (VHHs) against porcine epidemic diarrhea virus (PEDV) using yeast two-hybrid technology. The aim was to develop neutralizing nanobodies that can overcome the limitations of current vaccines and address the shortage of therapeutic antibodies for PEDV. By employing the PEDV spike (S) protein as bait, the researchers screened a synthetically constructed nanobody yeast library and identified seven neutralizing antibodies with potent antiviral activity. Moreover, combining these seven antibodies into a “cocktail” further enhanced their antiviral effects, highlighting their potential as a promising candidate for antibody-based therapeutics in preventing and treating PEDV.

## 1. Introduction

Porcine epidemic diarrhea (PED) is an acute diarrheal disease in pigs caused by the porcine epidemic diarrhea virus (PEDV) [1,2]. This disease is characterized by severe watery diarrhea, vomiting, dehydration, and high mortality rates, particularly in neonatal and weaned piglets, where the fatality rate is extremely high [3,4,5,6]. Over the past decades, PEDV has emerged as a major threat to the global pig industry, leading to significant mortality and severe economic losses, thereby impeding the industry’s healthy development [7,8,9,10,11,12]. Vaccination remains the primary method for preventing PED and controlling outbreaks. The development of novel vaccines, including subunit vaccines [13], viral vector vaccines [14,15], and bacterial vector vaccines [16,17], has broadened the options available for PED prevention and control. However, due to the high variability of the virus, the efficacy of existing commercial vaccines has been suboptimal [18]. At present, there are no effective preventive measures to manage and control the seasonal outbreaks of PED in neonatal piglets, leading to significant economic losses in the pig industry.

PEDV is an enveloped, single-stranded, positive-sense RNA virus belonging to the genus *Coronavirus* within the family *Coronaviridae*, order *Nidovirales* [19]. Its genome, approximately 28 kb in length, consists of seven open reading frames (ORF 1a/1b and ORF 2–6) encoding three non-structural proteins (pp1a, pp1b, ORF3) and four structural proteins: spike (S) protein (180–220 kDa), envelope (E) protein (27–32 kDa), membrane (M) protein (7 kDa), and nucleocapsid (N) protein (55–58 kDa) [20]. These structural proteins are essential for viral entry, assembly, and genome replication. The S protein, a homotrimeric class I fusion protein located on the viral surface, plays a crucial role in host recognition, receptor binding, and membrane fusion. During PEDV infection, the S protein is cleaved by host cell proteases, such as trypsin-like enzymes, into S1 (aa 1–789) and S2 (aa 790–1383) subunits, causing conformational changes [21,22]. The S1 subunit contains the N-terminal domain (NTD) and C-terminal domain (CTD), with the NTD responsible for sialic acid-binding activity and the CTD binding to cell surface receptors, such as aminopeptidase N (APN) [23]. The S2 subunit is responsible for mediating virus–cell membrane fusion [24]. The S protein also contains key neutralizing epitopes, including collagenase-26K equivalent (COE, aa 501–640), S1D (aa 640–794), and 2C10 (aa 1373–1379), among others [25]. These features make the S protein an excellent target for the development of vaccines that induce protective immunity against PEDV. Moreover, neutralizing antibodies targeting the S protein, when administered through passive immunization, can effectively protect neonatal piglets from PEDV infection [26,27,28,29].

Understanding the structure and function of antigenic epitopes is crucial for the development of immunotherapeutic drugs. Today, therapeutic antibody development in human medicine is well-established, with significant breakthroughs, particularly in cancer treatment. Moreover, there has been an increased focus on developing therapeutic antibodies for highly infectious diseases [30,31,32,33,34,35]. However, antibody research in veterinary medicine remains in its early stages, with initial progress made in detecting pig diseases and developing therapeutic antibodies [27,29,36]. Nanobodies (VHHs), due to their small size, ease of genetic manipulation, high specificity, and good solubility, have garnered considerable attention [37]. Currently, there are limited reports on nanobodies targeting pig diseases, and even fewer on those targeting PEDV. The commercialization and application of antibody therapeutics for pig diseases still face significant challenges [38]. With the increasing market demand, there is an urgent need to develop antibody therapeutics for pig diseases to complement vaccine-based prevention, address viral infections during the vaccine window period, and provide emergency treatments for early stage infections. Nanobodies, with their unique advantages, offer a promising direction for antibody research in this area.

To develop effective alternatives to current PEDV vaccines, we employed yeast two-hybrid technology with the S protein of PEDV as bait to screen a synthetic nanobody yeast library. This approach identified VHH gene sequences that interact with the S protein, which were subsequently expressed to produce PEDV S-VHH antibodies. The binding specificity of these nanobodies to PEDV and the S protein was confirmed via indirect immunofluorescence assay (IFA) and Western blotting, and their neutralizing efficacy was evaluated through neutralization experiments. Ultimately, seven neutralizing antibodies with in vitro antiviral activity were identified. Furthermore, we preliminarily predicted the specific binding sites of the PEDV S-VHHs on the S protein. Our findings lay the groundwork for the development of VHH-based therapeutics for the prevention and treatment of PEDV infection.

## 2. Materials and Methods

### 2.1. Cells, Viruses and Plasmids

Vero E6 and HEK293T cells were obtained from the Jiangsu Key Laboratory for High-Tech Research and Development of Veterinary Biopharmaceuticals. The cells were cultured in Dulbecco’s Modified Eagle’s Medium (DMEM) (Gibco, Carlsbad, CA, USA) enriched with 10% heat-inactivated fetal bovine serum (FBS) (GeminiBio, West Sacramento, CA, USA), 1% penicillin, and streptomycin. The cultures were maintained at 37 °C in a 5% CO_2_ incubator. FreeStyle 293-F cells (CTCC-001-0430) were procured from Meisen Cell Technology Co., Ltd. (Jinhua, China) and cultured in serum-free 293Pro CD 293 TT medium (BasalMedia Technology, Shanghai, China) supplemented with 1% penicillin and streptomycin. These cells were kept in suspension culture at 37 °C and 125 rpm in a shaking incubator with 8% CO_2_. Sf9 insect cells were maintained in our laboratory and cultured in serum-free SIM SF Expression Medium (Sino Biological, Beijing, China). These cells were grown in suspension at 27 °C in a CO_2_-free environment, with a passaging ratio of 1:5 every 3 to 4 days, ensuring a cell density of 1 × 10^6^ to 2 × 10^6^ cells/mL.

PEDV strain CV777 (GenBank accession number: AF353511.1) was propagated and preserved in our laboratory using Vero E6 cells. DMEM supplemented with 1% penicillin and streptomycin without FBS was used as the maintenance medium for virus amplification. PEDV strains JSXH-2021 and JXJA-2022 were isolated and preserved in our laboratory. For amplification, the virus maintenance medium was DMEM without FBS, supplemented with 7.5 µg/mL trypsin (Sigma-Aldrich, St. Louis, MO, USA).

The eukaryotic expression plasmid for PEDV S-VHH (pCDNA3.1-VHH, containing a 6 × His tag at the C-terminus) was synthesized by Azenta Life Sciences (Suzhou, China). The insect baculovirus expression plasmid pFastBac1-PEDV-S (containing a 3 × FLAG tag at the C-terminus) was synthesized by Jiyu Biotechnology Co., Ltd. (Changzhou, China). The bait plasmid pGBKT7-PEDV-S and the artificially synthesized nanobody yeast library plasmid (pGADT7-VHH) were provided by Ruiyuan Biotechnology (Nanjing, China). The S gene sequences mentioned above are all derived from the PEDV-JSXH-2021 strain.

### 2.2. Propagation of PEDV and Acquisition of S Gene Sequences

#### 2.2.1. Propagation of PEDV

Vero E6 cells were passaged and cultured until they reached over 90% confluence within 24 h and then infected with PEDV. Prior to infection, the cell surface was washed twice with an appropriate volume of PBS, followed by the addition of a virus maintenance medium. The cells were infected with PEDV at a multiplicity of infection (MOI) of 0.01. The culture flask was gently shaken and incubated at 37 °C with 5% CO_2_. The cells were observed daily, and when over 70% exhibited cytopathic effects (CPE), the virus supernatant was collected. The infected Vero E6 cell cultures were subjected to three cycles of freeze–thawing to lyse the cell membranes and release the viral particles. The mixture was centrifuged at 4000× *g* for 10 min, and the supernatant was collected, filtered through a 0.2 μm filter, aliquoted, and stored at −80 °C for future use.

#### 2.2.2. TCID_50_ Assay

Vero E6 cells were seeded into a 96-well cell culture plate and cultured until they reached over 90% confluence within 24 h. The cells were washed twice with PBS before infection to determine the TCID_50_. The virus stock solution was serially diluted in virus maintenance medium in 10-fold, and 100 μL of each dilution was added to the 96-well plate, with eight replicates for each dilution. The plate was incubated in a cell culture incubator for 1 h, allowing the cells to be exposed to the virus. After the incubation, the virus solution was aspirated, and the cells were washed twice with PBS. Then, 100 μL of virus maintenance medium was added to each well, and the plate was returned to the incubator at 37 °C with 5% CO_2_. The number of wells showing CPE was recorded daily, and the TCID_50_ value was calculated using the Reed–Muench method.

#### 2.2.3. Amplification and Sequencing of the PEDV S Gene

RNA from the PEDV-JSXH-2021 strain was extracted using the FastPure Viral DNA/RNA Mini Kit (Vazyme, Nanjing, China) and converted into cDNA using the HiScript IV 1st Strand cDNA Synthesis Kit (Vazyme, Nanjing, China). The cDNA was then utilized as a template to amplify the S gene sequence of the PEDV-JSXH-2021 strain, employing the 2 × Phanta Flash Master Mix (Vazyme, Nanjing, China). The PEDV S gene sequence was amplified in two segments: S1 and S2. The primers for amplifying the S1 region were S1-2F (5′-TGAAGAATGGTAAGTTGCTAGTGC-3′) and S1-7R (5′-TGGGTGAGTAATTGTTTACAACG-3′), yielding an amplification product size of 2550 bp. For the S2 region, the primers were L-PEDV-S2-F1 (5′-GGGAGTTGCCTGGTTTCTTC-3′) and L-PEDV-S2-R3 (5′-CATTCACTGCACGTGGACC-3′), with an amplification product size of 1979 bp. The amplified S1 and S2 products were purified using the E.Z.N.A. Gel Extraction Kit (Omega Bio-tek, Norcross, GA, USA) and subsequently cloned into the T-vector (Solarbio Science & Technology, Beijing, China) for sequencing analysis.

### 2.3. Screening of PEDV S-VHHs

The bait plasmid was transformed into the yeast strain AH109 and tested for self-activation. Both pGBKT7-PEDV-S and the pGBKT7 control plasmids were transformed into AH109 using the following protocol. Single colonies of AH109 were picked from a YPDA plate and inoculated into 4 mL of YPDA liquid medium, then incubated at 30 °C with shaking at 225 rpm for 18–20 h until the OD_600_ was more than 1.5. The culture was then transferred to 50 mL of YPDA liquid medium to an initial OD_600_ of 0.2 and incubated at 30 °C with shaking at 225 rpm for 4–5 h until the OD_600_ reached 0.6. The yeast cells were collected by centrifugation at room temperature at 4000 rpm for 5 min. The pellet was resuspended in 20 mL of sterile water, mixed, and centrifuged again at room temperature at 4000 rpm for 5 min, after which the supernatant was discarded. The pellet was resuspended in 5 mL of 0.1 M LiAc, mixed, and centrifuged at 4000 rpm for 5 min. The supernatant was discarded, and the pellet was resuspended in 500 μL of 0.1 M LiAc, mixed, and aliquoted into centrifuge tubes, with 50 μL for each transformation. For each transformation, 240 μL of 50% PEG3350, 36 μL of 1 M LiAc, 5 μL of ssDNA (20 mg/mL), and 5 μL of pGBKT7-PEDV-S or pGBKT7 plasmid were added to the tube. The mixture was gently mixed by pipetting or vigorously shaken for about 1 min until fully combined. The tubes were incubated in a 30 °C water bath for 30 min, followed by heat shock at 42 °C for 25 min, and then returned to the 30 °C water bath for another 30 min. The yeast cells were collected by centrifugation at room temperature at 4000 rpm for 5 min, and the supernatant was discarded. Each transformation product was resuspended in 200 μL of sterile water, mixed gently, and spread onto SD-T (SD/-Trp) plates, which were incubated at 30 °C for 3–4 days. Three colonies were randomly picked and transferred to SD-T, SD-TH (SD/-Trp/-His), SD-THA (SD/-Trp/-His/-Ade), and SD-THA + X-α-gal plates and incubated at 30 °C for 3–5 days, with pGBKT7 as a negative control.

For library screening, AH109 yeast strains containing the pGBKT7-PEDV-S bait plasmid were prepared for transformation with the library plasmid pGADT7-VHH. The transformed yeast was spread onto 20 SD-TLH (SD/-Trp/-Leu/-His) selection plates and incubated at 30 °C for 3–7 days to observe colony growth. Colonies were picked and transferred to SD-TLHA (SD/-Trp/-Leu/-His/-Ade) + X-α-gal selection plates for further incubation for 3–5 days.

Positive yeast clones were identified and sequenced. The Yeast Colony Rapid Detection Kit (Ruiyuan Biotechnology, Nanjing, China) was adopted to amplify the sequences of positive yeast clones with primers T7-F (TAATACGACTCACTATAGG) and BD-R (GAATTAGCTTGGCTGCAAGC). Eight distinct VHH gene sequences were identified utilizing DNA sequencing and BLAST analysis (Table S1).

To verify the positive yeast clones, they were diluted in sterile water and spotted onto SD-TL, SD-TLH, SD-TLHA, and SD-TLHA + X-α-gal deficiency plates. The plates were incubated at 30 °C for 3–4 days to observe colony growth.

### 2.4. Expression and Purification of PEDV S-VHHs

#### 2.4.1. Expression of PEDV S-VHHs in Eukaryotic Cells

PEDV S-VHHs were expressed in HEK293T cells and FreeStyle 293-F cells, respectively.

Once HEK293 T cells upon reached 70–90% confluence, the cells in a 6-well plate were transfected with 2 μg of pcDNA3.1-VHH plasmid using X-tremeGENE HP DNA Transfection Reagent (Roche, Basel, Switzerland). After 72 h, the culture supernatant and cell pellet were collected separately to detect the expression of PEDV S-VHHs.

The FreeStyle 293-F cell transfection was performed when the cell density in a 50 mL suspension culture reached approximately 2 × 10^6^ cells/mL and the cell viability exceeded 90%. Two clean, sterile centrifuge tubes were prepared—one containing the pcDNA3.1-VHH plasmid and the other containing 100 μL of Lipo293 transfection reagent (Beyotime Biotechnology, Shanghai, China). The solutions were gently mixed by pipetting. The medium containing the DNA was gently added to the medium containing the Lipo293 transfection reagent, and the mixture was gently mixed again by pipetting. After incubating the mixture at room temperature for 15 min, 2.5 mL of the Lipo293-DNA mixture was added to the 50 mL suspension culture of FreeStyle 293-F cells. The cells were incubated in a 37 °C shaker incubator at 125 rpm with 8% CO_2_ for 72 h. Following incubation, the cells were harvested to detect target protein expression.

#### 2.4.2. Purification of PEDV S-VHHs

PEDV S-VHHs were purified using HisSep Ni-NTA MagBeads (Yeasen Biotechnology, Shanghai, China). Seventy-two hours post-transfection, FreeStyle 293-F cells were collected and lysed in Lysis Buffer at a ratio of 1:10 (W/V), supplemented with 1 mM PMSF. Cell disruption was performed on ice using ultrasonic treatment until the lysate appeared mostly clear. Lysis Buffer containing 8 M urea was added to the lysate for a final volume of 40 mL, and the mixture was incubated overnight at 4 °C with continuous mixing. The next day, the mixture was centrifuged at 15,000× *g* for 15 min at 4 °C, and the supernatant was transferred to a new tube. Magnetic beads were then added to the supernatant and incubated overnight at 4 °C with gentle inversion. After five washes with Wash Buffer containing 8 M urea, the protein was eluted using Elution Buffer (also containing 8 M urea). The eluted protein was subjected to desalting using a desalting column (Beyotime Biotechnology, Shanghai, China) for refolding. The final refolded PEDV S-VHHs were adjusted to a concentration of 200 ng/μL for subsequent experiments and stored at −80 °C until use.

#### 2.4.3. Western Blotting

To detect the expression of PEDV S-VHHs in FreeStyle 293-F cells, 1 mL of cells was harvested 72 h post-transfection. Total protein was extracted utilizing RIPA lysis buffer supplemented with 1% PMSF (Beyotime Biotechnology, Shanghai, China). SDS-PAGE was performed utilizing a 15% pre-cast gel (Yeasen Biotechnology, Shanghai, China). Following electrophoresis, the proteins were transferred onto a PVDF membrane at a constant current of 300 mA for 2 h. After transfer, the membrane was blocked at room temperature for 1.5 h and then incubated overnight at 4 °C with a mouse anti-His tag monoclonal antibody (Solarbio Science Technology, Beijing, China; 1:1000) as the primary antibody. After three washes with TBST, the membrane was incubated for 1 h at room temperature with a goat anti-mouse HRP-conjugated secondary antibody (Solarbio Science Technology, Beijing, China; 1:10,000). The membrane was then washed three times with TBST before undergoing color development.

### 2.5. Specific Identification of PEDV S-VHHs

#### 2.5.1. Detection of Binding Specificity of PEDV S-VHHs and the PEDV S Protein

Construction of recombinant bacmid: The recombinant plasmid pFastBac1-PEDV-S (containing a 3 × Flag tag at the C-terminus) was transformed into DH10Bac competent cells using the heat shock method. Blue-white colony screening was performed on LB plates containing kanamycin, gentamicin, tetracycline, IPTG, and X-gal, which were incubated in the dark at 37 °C for 48 h. Monoclonal white colonies were selected and cultured at 37 °C with shaking for 16–24 h to extract the recombinant bacmid (Bacmid-PEDV-S).

Amplification of recombinant baculovirus: Sf9 cells in the logarithmic growth phase with a viability greater than 90% were seeded at a density of 2 × 10^6^/well in a 6-well plate. Bacmid-PEDV-S was mixed uniformly with the transfection reagent Cellfectin II Reagent (Thermo Fisher Scientific, Waltham, MA, USA) according to the manufacturer’s instructions. After a 30 min incubation at room temperature, the mixture was added to the 6-well plate and incubated at 27 °C for 6 h. The medium was then replaced with serum-free culture medium and maintained until noticeable cytopathic effects were observed, at which point the supernatant was collected as the P1 generation recombinant baculovirus (rBV-PEDV-S). Subsequent passages resulted in the P3 generation rBV-PEDV-S.

Expression of recombinant S protein and detection of binding specificity of PEDV S-VHHs: Sf9 cells were seeded at a density of 2 × 10^6^/well in a 6-well plate, and P3 generation rBV-PEDV-S was used to infect the Sf9 cells at a multiplicity of infection (MOI) of 0.1. After 48 h of incubation at 27 °C, the cells were fixed with 4% paraformaldehyde for IFA. Diluted PEDV S-VHHs served as the primary antibody, a mouse anti-His tag monoclonal antibody was used as the secondary antibody, and a goat anti-mouse IgG (H+L) conjugated with Alexa Fluor 488 was used as the tertiary antibody. Infected Sf9 cells with a mouse anti-Flag tag served as the positive control, while uninfected Sf9 cells served as the mock control.

#### 2.5.2. Detection of Binding Specificity of PEDV S-VHHs and the PEDV

Vero E6 cells were infected with PEDV-JSXH-2021 at an MOI of 0.01. After 48 h, the cells were removed from the incubator, and the culture medium was discarded. The cells were washed twice with PBS and then fixed with 4% paraformaldehyde at 4 °C for at least 20 min, followed by three washes with PBS. Immunofluorescence blocking buffer was added and incubated at room temperature for 1 h. Purified PEDV S-VHHs, diluted to the appropriate concentration, were used as the primary antibody and incubated overnight at 4 °C. The cells were washed three times with PBS, each lasting 5 min. A mouse anti-His tag monoclonal antibody (diluted 1:500) was added and incubated for 1 h at room temperature. After incubation, the cells were washed three more times with PBS. A goat anti-mouse IgG (H+L) conjugated with Alexa Fluor 555 antibody (Thermo Fisher Scientific, Waltham, MA, USA; diluted 1:400) was added and incubated in the dark at room temperature for 1 h, followed by three washes with PBS. Finally, a DAPI anti-fade mounting medium (Beyotime Biotechnology, Shanghai, China) was added to cover the cells, which were then stored in the dark at 4 °C.

### 2.6. Neutralizing Activity of PEDV S-VHHs Against PEDV

Vero E6 cells were pre-seeded in a 96-well culture plate to achieve 70–80% confluency for later use. In a separate 96-well plate, serial dilutions of the VHHs were prepared, with four replicate wells for each dilution. To each well, 50 µL of the diluted virus solution (containing 200 TCID_50_ of the virus suspension) was added, mixed thoroughly, and incubated at 37 °C with 5% CO_2_ for 60 min. Negative control (PEDV N protein antibody), virus control, and normal cell control groups were also established. The virus control group included four different virus concentrations: 200 TCID_50_ (expected to exhibit a full cytopathic effect), 20 TCID_50_, 2 TCID_50_, and 0.2 TCID_50_ (expected to show no cytopathic effect). After incubation, 100 µL of the virus-VHH antibody mixture was added to each well of the 96-well plate containing Vero E6 cells. The plate was then incubated at 37 °C with 5% CO_2_ for 60 min. Subsequently, we changed the medium and added 200 µL of virus maintenance medium to each well, continuing to culture at 37 °C. After 5 to 7 days, we performed IFA to determine whether any lesions had developed in each well, recording and analyzing the neutralization effects of each VHH. The experiment should be repeated three times.

### 2.7. Prediction of Antibody Binding Sites

The three-dimensional structures of the PEDV S protein and PEDV S-VHHs were modeled using AlphaFold2 software v1.5.3, and the corresponding PDB files were obtained. These PDB files were analyzed using the docking software Hdocklite v1.1 to predict interactions between the PEDV S protein and the VHHs. Confidence scores were generated, with higher scores indicating a greater likelihood of interaction. The predicted binding sites between the PEDV S protein and the VHHs were further analyzed using the PDBePISA website. Finally, the interaction sites were annotated and refined using PyMol 2.5 software to provide a detailed visualization of the protein–protein interactions.

### 2.8. Statistical Analysis

All values were presented as means ± SEM. All experiments were performed in triplicate.

## 3. Results

### 3.1. Screening of PEDV S-VHHs

**Figure 1:**
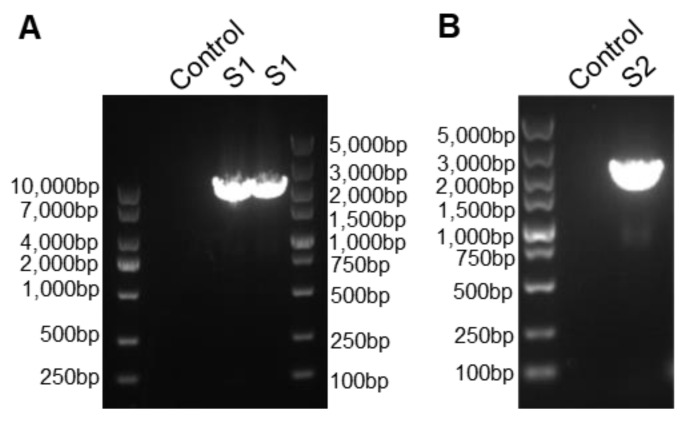
Amplification results of the S1 and S2 gene from the porcine epidemic diarrhea virus (PEDV) strain JSXH-2021. (**A**) PCR amplification results of the S1 gene from the PEDV-JSXH-2021 strain. cDNA from the PEDV-JSXH-2021 strain served as the template, using primers S1-2F (5′-TGAAGAATGGTAAGTTGCTAGTGC-3′) and S1-7R (5′-TGGGTGAGTAATTGITTACAACG-3′) to amplify the S1 gene. An amplification reaction without template was included as a negative control. (**B**) PCR amplification results of the S2 gene from the PEDV-JSXH-2021 strain. The primers L-PEDV-S2-F1 (5′-GGGAGTTGCCTGGTTTCTTC-3′) and L-PEDV-S2-R3 (5′-CATTCACTGCACGTGGACC-3′) were used to amplify the S2 gene, with a reaction lacking template serving as a negative control.

This study aimed to utilize the S gene of the PEDV-JSXH-2021 strain as bait to screen a nanobody library for VHHs. To accomplish this, it was essential to first obtain the complete sequence of the S gene. Given the challenges associated with amplifying the S gene, we separately amplified the S1 and S2 genes. As demonstrated in Figure 1, we successfully amplified a 2550 bp fragment containing the S1 gene and a 1979 bp fragment containing the S2 gene. Following amplification, sequencing and assembly were conducted to obtain the complete sequence of the PEDV-JSXH-2021 S gene.

**Figure 2:**
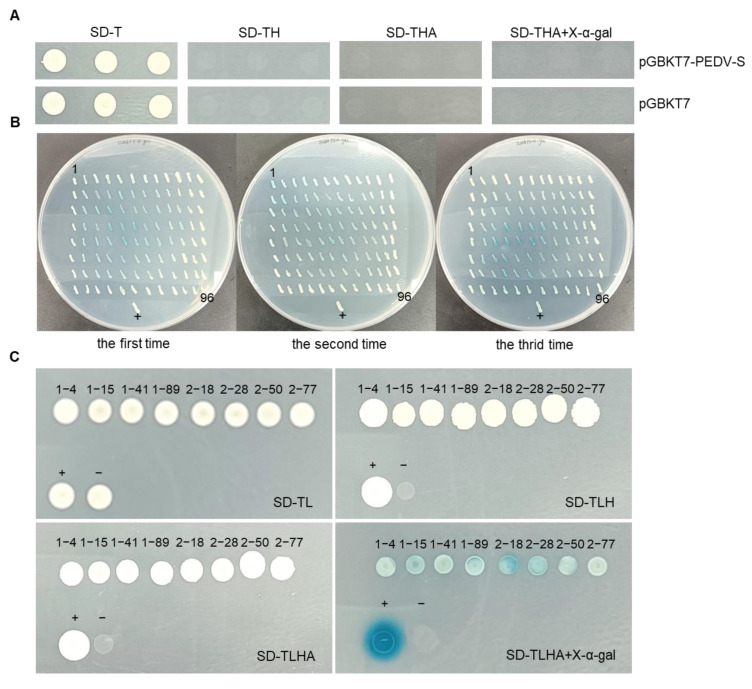
Selection of nanobodies interacting with pGBKT7-PEDV-S through yeast two-hybrid screening. (**A**) Transformation of the bait plasmid pGBKT7-PEDV-S into the receptor yeast strain AH109, followed by self-activation detection. (**B**) Screening of positive yeast clones on SD-TLHA + X-α-gal selection plates. (+) serves as a positive control: pGADT7-largeT + pGBKT7-p53. (**C**) Verification of positive yeast clones by plasmid rescue. (+) serves as a positive control with pGADT7-largeT+pGBKT7-p53; (−) serves as a negative control with pGADT7-largeT + pGBKT7-laminC.

This sequence was then inserted into the pGBKT7 vector to construct the bait vector, pGBKT7-PEDV-S. Using this bait, the nanobody yeast library pGADT7-VHH was screened via yeast two-hybrid screening. Initially, the pGBKT7-PEDV-S and pGBKT7 vectors were transformed into AH109 yeast strains for self-activation testing. As shown in Figure 2A, the negative control (pGBKT7) grew only on SD-T plates and did not grow on SD-TH, SD-THA, or SD-THA + X-α-gal plates. The experimental group (pGBKT7-PEDV-S) exhibited similar growth patterns to the control, confirming that pGBKT7-PEDV-S does not exhibit self-activation. Next, AH109 yeast strains containing the pGBKT7-PEDV-S bait plasmid were prepared as competent cells, and the pGADT7-VHH library plasmid was introduced. The selection was performed on SD-TLH screening plates, resulting in 288 positive yeast clones (Figure 2B). These target sequences were amplified, sequenced, and analyzed, yielding eight distinct antibody amino acid sequences, as listed in Table S1. These eight positive clones containing antibodies were further validated by re-transformation. As shown in Figure 2C, the positive control (pGADT7-largeT + pGBKT7-p53) grew on SD-TL, SD-TLH, SD-TLHA, and SD-TLHA + X-α-gal plates, displaying blue coloration on SD-TLHA + X-α-gal plates. In contrast, the negative control (pGBKT7) grew only on SD-TL plates. All eight positive clones from the screening process grew normally on SD-TL, SD-TLH, and SD-TLHA plates and exhibited blue color on SD-TLHA + X-α-gal plates. Thus, through yeast two-hybrid screening, eight VHH amino acid sequences that interact with pGBKT7-PEDV-S were identified, and they were designated as PEDV S-VHHs, with the following labels: VHH1–4, VHH1–15, VHH1–41, VHH1–89, VHH2–18, VHH2–28, VHH2–50, and VHH2–77.

### 3.2. Eukaryotic Expression and Purification of Selected PEDV S-VHHs

**Figure 3:**
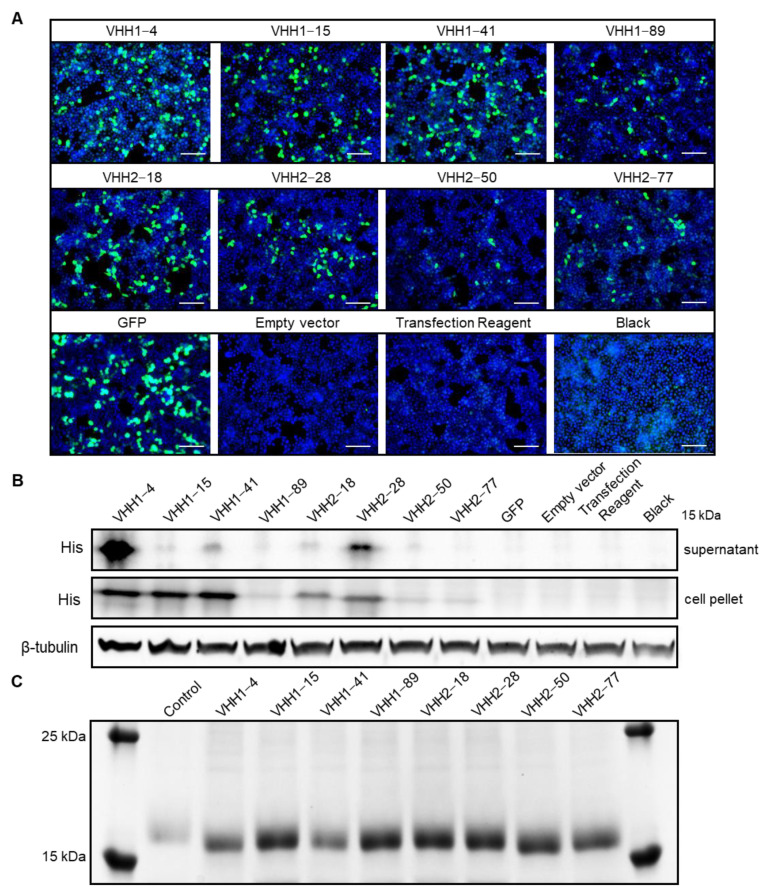
Eukaryotic expression, identification, and purification of PEDV S-VHHs. (**A**) IFA detection of PEDV S-VHH expression in HEK293T cells. The pcDNA3.1-VHH plasmids were transfected into HEK293T cells, and expression of VHH1–4, VHH1–15, VHH1–41, VHH1–89, VHH2–18, VHH2–28, VHH2–50, and VHH2–77 was detected 72 h post-transfection using a mouse anti-His tag monoclonal antibody. The group transfected with the pEGFP-N1 plasmid served as the positive control; the group transfected with the empty vector served as the negative control; the group treated only with the transfection reagent was used to assess the toxicity of the reagent on the cells; the untreated cell culture group served as the blank control. Green color represents the VHHs, except in the GFP images, where green color represents the GFP; blue color represents nuclei. Scale bar = 100 µm. (**B**) Western blotting analysis of PEDV S-VHH expression in HEK293T cells. Supernatants and cell pellets from each group of cultured cells were collected for analysis. A mouse anti-His tag monoclonal antibody was used to detect VHH1–4, VHH1–15, VHH1–41, VHH1–89, VHH2–18, VHH2–28, VHH2–50, and VHH2–77. The expected molecular weight of the target proteins was approximately 15 kDa. (**C**) SDS-PAGE analysis of purified PEDV S-VHH proteins. The purification of PEDV S-VHH proteins from FreeStyle 293-F cells is shown, with control samples consisting of cell pellets from those transfected with an empty vector.

To confirm whether these VHHs specifically recognize PEDV and exhibit neutralizing activity, the VHHs were expressed and purified at first. To produce the selected antibodies, VHHs expression plasmids (pcDNA3.1-VHH) were constructed and transfected into HEK293T cell lines to evaluate their expression. After 72 h, the HEK293T cells were fixed with 4% paraformaldehyde for IFA detection. Additionally, cell pellet samples were collected for Western blotting. The group transfected with the pEGFP-N1 plasmid served as the positive control, while the group transfected with the empty vector served as the negative control. Additionally, the group treated only with the transfection reagent was used to assess the toxicity of the reagent on the cells, and the untreated cell group served as the blank control. As shown in Figure 3A,B, both IFA and Western blotting confirmed the successful expression of all eight PEDV S-VHHs. Among them, VHH1–4, VHH1–15, VHH1–41, VHH2–18, and VHH2–28 demonstrated relatively higher expression levels, whereas VHH1–89, VHH2–50, and VHH2–77 showed lower expression. For large-scale production, the antibody expression plasmids were transfected into FreeStyle 293-F cells cultured in suspension (Figure S2). The cells were harvested and disrupted by sonication, and the PEDV S-VHHs were purified. SDS-PAGE analysis of the purified antibodies (Figure 3C) revealed single, distinct protein bands near the 15 kDa marker for all eight VHHs, matching the expected sizes and confirming successful purification. After desalting and refolding, the concentrations of the purified VHHs were determined as follows: 0.914 µg/µL, 0.271 µg/µL, 0.593 µg/µL, 0.359 µg/µL, 0.228 µg/µL, 0.596 µg/µL, 0.322 µg/µL, and 0.386 µg/µL, respectively. These results demonstrate that functional VHH antibodies were successfully produced through recombinant expression and purification, yielding sufficient quantities for subsequent evaluations of binding specificity and neutralizing activity.

### 3.3. Binding Specificity of PEDV S-VHHs to PEDV and Its S Protein

**Figure 4:**
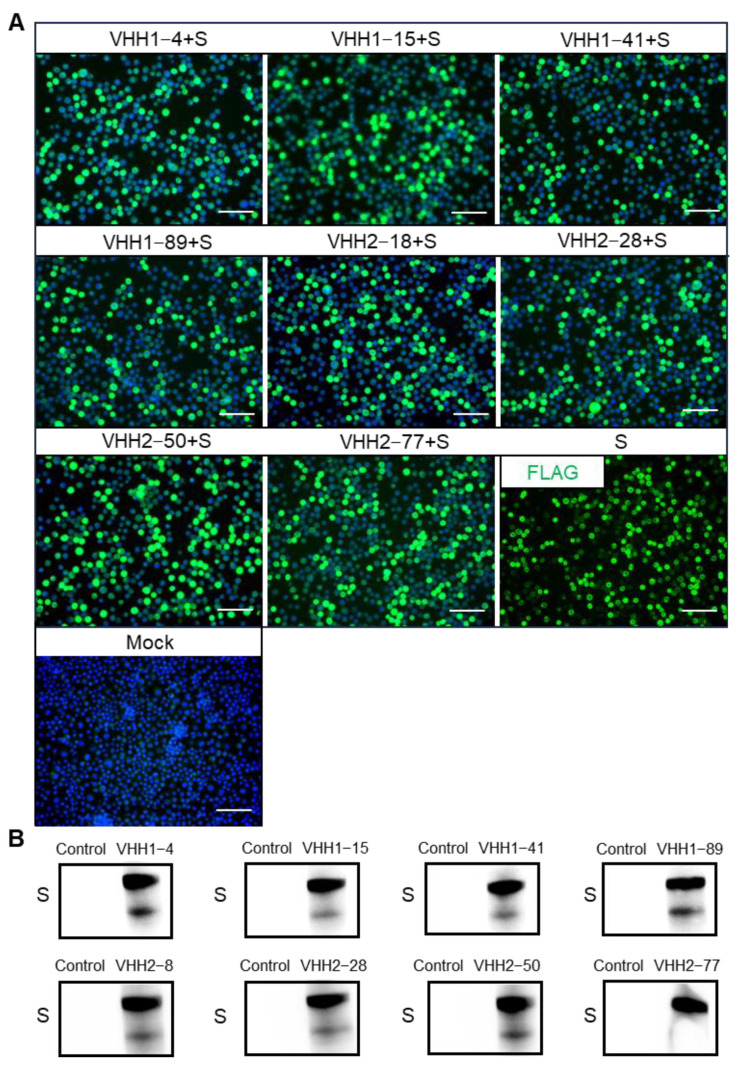
Binding ability of PEDV S-VHHs to PEDV S protein. (**A**) IFA detection of specific binding of PEDV S-VHHs to the S protein. The P3 generation rBV-PEDV-S was used to infect Sf9 cells at a MOI of 0.1, and after 48 h, the cells were prepared for IFA detection. The PEDV S-VHHs served as the primary antibodies, with a mouse anti-His tag monoclonal antibody as the secondary antibody and a goat anti-mouse IgG (H+L) conjugated with Alexa Fluor 488 as the tertiary antibody. Sf9 cells not infected with rBV-PEDV-S served as the mock control. The Sf9 cells infected with the rBV-PEDV-S were labeled with a mouse anti-Flag tag as the positive control. Green color represents the S protein; blue color represents nuclei. Scale bar = 100 µm. (**B**) Western blotting analysis of the binding of PEDV S-VHHs to the S protein. Supernatants from sonicated Sf9 cells infected with recombinant baculovirus for 48 h were analyzed. The PEDV S-VHHs were used as the primary antibodies, and a mouse anti-His tag (HRP) monoclonal antibody was used as the secondary antibody. Sf9 cells not infected with the recombinant baculovirus were used as the control.

The binding specificity of the PEDV S-VHHs was evaluated. The activity of these nanobodies in binding to the PEDV S protein was assessed via IFA and Western blotting. We employed the following experimental strategy: the P3 generation rBV-PEDV-S was used to infect Sf9 cells at a multiplicity of infection (MOI) of 0.1, and after 48 h, the cells were prepared for IFA detection. Specifically, diluted PEDV S-VHHs were used as the primary antibody, a mouse anti-His tag monoclonal antibody served as the secondary antibody, and a goat anti-mouse IgG (H+L) conjugated with Alexa Fluor 488 was used as the tertiary antibody. Infected Sf9 cells labeled with a mouse anti-Flag tag served as the positive control, while uninfected Sf9 cells served as the mock control. As shown in Figure 4A, IFA analysis demonstrated that the refolded VHH1–4, VHH1–15, VHH1–41, VHH1–89, VHH2–18, VHH2–28, VHH2–50, and VHH2–77 all successfully detected the PEDV S protein expressed in Sf9 cells. Additionally, Western blot analysis of disrupted Sf9 cells expressing the S protein revealed that all eight nanobodies specifically detected a band of approximately 180 kDa, confirming their ability to bind the PEDV S protein (Figure 4B).

**Figure 5:**
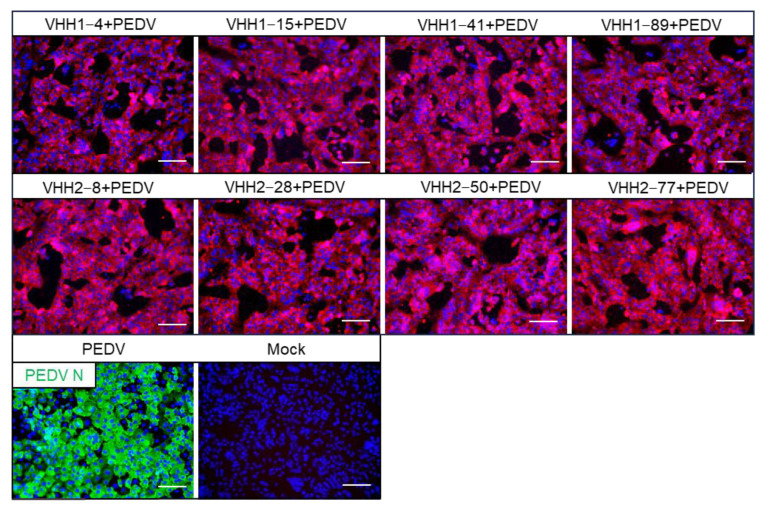
IFA analysis of the binding of PEDV S-VHHs to PEDV. Vero E6 cells infected with the PEDV-JSXH-2021 strain (MOI = 0.01) for 48 h were used for detection. PEDV S-VHHs were employed as the primary antibodies, with a mouse anti-His tag monoclonal antibody as the secondary antibody, and a goat anti-mouse IgG (H+L) conjugated with Alexa Fluor 555 as the tertiary antibody. Vero E6 cells not infected with PEDV-JSXH-2021 served as the mock control. The Vero E6 cells infected with the PEDV-JSXH-2021 strain were labeled with a mouse anti-PEDV N polyclonal antibody as the positive control. Red color represents the PEDV; green color represents the PEDV N protein; blue color represents nuclei. Scale bar = 100 µm.

To further assess the specificity of PEDV S-VHHs in recognizing the PEDV virus, IFA was performed on Vero E6 cells infected with the PEDV-JSXH-2021 strain. The results showed that all eight VHHs (VHH1–4, VHH1–15, VHH1–41, VHH1–89, VHH2–18, VHH2–28, VHH2–50, and VHH2–77) specifically labeled PEDV-infected Vero E6 cells, while no labeling was observed in uninfected control cells, confirming that PEDV S-VHHs can specifically recognize and bind to the PEDV strain (Figure 5). In conclusion, the VHHs identified in our screening specifically recognize and bind to the PEDV S protein, highlighting their potential diagnostic applications.

### 3.4. Neutralizing Activity of PEDV S-VHHs Against PEDV

Neutralization assays were conducted to evaluate the neutralizing activity of the eight selected PEDV S-VHHs against PEDV. The refolded PEDV S-VHHs were standardized to a concentration of 200 ng/µL and serially diluted to assess their neutralizing capacity, ensuring four replicates for each dilution. Specifically, the diluted VHHs were mixed with an equal volume of 200 TCID_50_/mL PEDV-JSXH-2021 and incubated at 37 °C for 60 min, using PEDV N protein antibody as a negative control. After incubation, the PEDV-VHH mixture was added to a 96-well plate seeded with Vero E6 cells, followed by another 60 min of incubation. The virus maintenance medium was then replaced. After 5 to 7 days, the neutralization effects of each VHH were assessed by analyzing the number of wells exhibiting lesions through IFA. The neutralization assays were repeated three times.

**Figure 6:**
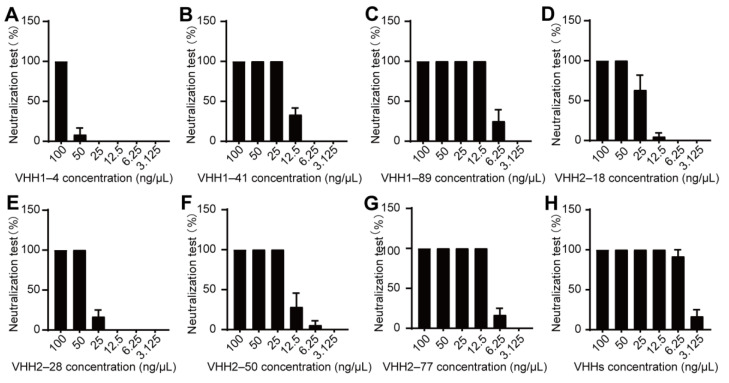
Neutralizing activity of PEDV S-VHHs against PEDV. After standardizing the concentration of PEDV S-VHHs to 200 ng/µL, they were serially diluted for neutralization assays, with four replicates for each dilution. The antiviral activity of the VHHs was assessed according to the neutralization experimental protocol; error bars represent the mean ± SEM, *n* = 3. (**A**–**G**) Neutralizing activity of VHH1–4, VHH1–41, VHH1–89, VHH2–18, VHH2–28, VHH2-50, and VHH2-77 against the PEDV-JSXH-2021 strain. (**H**) Neutralizing activity of a mixture of VHH1–4, VHH1–41, VHH1–89, VHH2–18, VHH2–28, VHH2–50, and VHH2–77 against the PEDV-JSXH-2021 strain.

As shown in Figure 6A–G and Table S2, VHH1–4, VHH1–41, VHH1–89, VHH2–18, VHH2–28, VHH2–50, and VHH2–77 exhibited neutralizing capability against the PEDV-JSXH-2021 strain, completely neutralizing viral infection at concentrations of 100 ng/µL, 25 ng/µL, 12.5 ng/µL, 50 ng/µL, 50 ng/µL, 25 ng/µL, and 12.5 ng/µL, respectively. In contrast, VHH1–15 did not demonstrate any neutralizing activity. These findings indicate that VHH1–4, VHH1–41, VHH1–89, VHH2–18, VHH2–28, VHH2–50, and VHH2–77 possess the ability to combat PEDV infection in vitro.

To further investigate whether a combination of these nanobodies could enhance the neutralizing effect, a “cocktail” mixture of the seven neutralizing VHHs was prepared—with each VHH at a concentration of approximately 30 ng/µL—to analyze their effect on viral neutralization. The results indicated that this combination of VHHs could completely inhibit the infection of PEDV-JSXH-2021 at a concentration of 12.5 ng/µL. Notably, at a concentration of 6.25 ng/µL, the protection rate remained over 90%, demonstrating superior antiviral effects compared to the use of any single VHH alone (Figure 6H). These findings indicate that our study successfully generated VHHs with neutralizing activity in vitro. Furthermore, the combined application of VHHs offers new insights for developing more effective treatments against PEDV.

### 3.5. Prediction of Binding Sites Between PEDV S-VHHs and the S Protein

**Figure 7:**
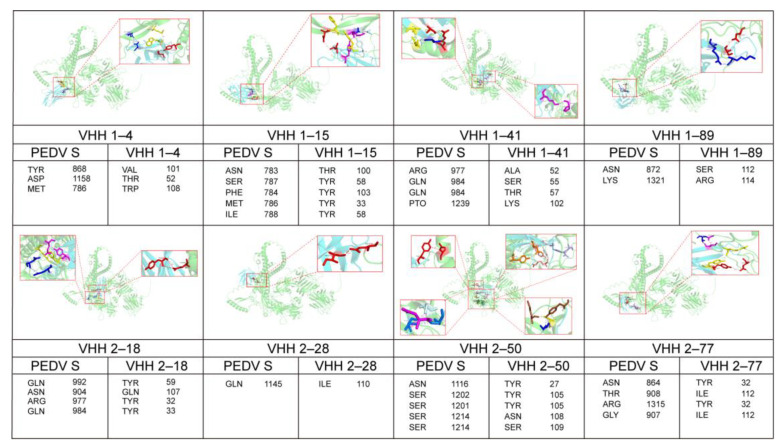
Prediction of interactions between PEDV S-VHHs and the S protein. The predicted binding sites and interactions between PEDV S-VHHs (VHH1–4, VHH1–15, VHH1–41, VHH1–89, VHH2–18, VHH2–28, VHH2–50, and VHH2–77, cyan) and the S protein (green) are shown. The binding sites are highlighted in red boxes, and all interactions depicted are hydrogen bonds. Specific amino acid residues at the binding sites are listed below each corresponding diagram. The S protein sequence is derived from the PEDV-JSXH-2021 strain.

To explore the interactions between the PEDV S-VHHs and the S protein (derived from the PEDV-JSXH-2021 strain), bioinformatics tools were employed for 3D structure modeling, molecular docking, and binding site prediction. Figure 7 illustrates the interactions between the eight selected PEDV S-VHHs and the S protein, highlighting the predicted binding sites within the CDRs of the VHHs and their target sites on the S protein. The results confirm that all VHHs—VHH1–4, VHH1–15, VHH1–41, VHH1–89, VHH2–18, VHH2–28, VHH2–50, and VHH2–77—specifically interact with the S protein. These findings are consistent with our earlier experimental results, reinforcing the specificity of these nanobodies for the S protein. Figure 7 provides detailed binding site information, showing that all predicted interactions involve hydrogen bonds. Notably, the CDRs of all eight VHHs predominantly bind to the S2 region of the S protein, which plays a crucial role in viral fusion and entry.

## 4. Discussion

Currently, porcine epidemic diarrhea virus (PEDV) remains one of the most significant viral pathogens affecting pigs, posing substantial challenges to the industry. Vaccination is the primary preventive measure against PEDV, but due to the virus’s high variability, existing vaccines have struggled to induce effective protective antibodies in pigs. Moreover, no effective therapeutic interventions are available once an outbreak occurs. These limitations lead to considerable economic losses. Therefore, there is an urgent need to develop new immune agents to combat PEDV, with antibodies playing a pivotal role in targeted treatments against the virus.

This study focused on the development of neutralizing antibodies against PEDV, specifically targeting the S protein, which plays a crucial role in viral entry into host cells. We screened for potential nanobodies with neutralizing activity against PEDV using the S gene sequence from the PEDV-JSXH-2021 strain [22,39]. This strain was isolated from a pig farm in Xinghua, Jiangsu Province, in 2021. The complete S gene sequence was obtained by amplifying the S1 and S2 gene segments, followed by sequencing and assembly. Phylogenetic analysis reveals that the PEDV-JSXH-2021 strain belongs to the G1b type, which remains prevalent in pig farms today [40]. The S gene was cloned into the pGBKT7 vector and used as bait in screening a nanobody yeast library, from which we successfully obtained a series of nanobodies with PEDV-neutralizing potential. Validation with the homologous PEDV-JSXH-2021 strain confirmed that eight nanobodies (VHHs) could specifically bind to this strain. To further evaluate the binding specificity of these VHHs to heterologous PEDV strains, we tested them against the CV777 (G1a type) and PEDV-JXJA-2022 (G2 type, also isolated in our laboratory) strains using IFA. The results showed that VHH1–15, VHH1–89, VHH2–18, and VHH2–28 specifically recognized the CV777 strain (Figure S3A), while VHH1–4, VHH1–15, VHH1–89, VHH2–18, VHH2–28, and VHH2–77 recognized and bound to the PEDV-JXJA-2022 strain (Figure S3B). However, it is important to note that effective antigen binding does not necessarily guarantee neutralization of the target virus. Future research should focus on validating the neutralizing effects of these VHHs against heterologous PEDV strains to evaluate their broader application potential. The seven VHHs that demonstrate neutralizing activity against the PEDV-JSXH-2021 strain show promise for further development as therapeutic agents for treating homologous PEDV infections.

Previous studies [41,42] have shown that when antibodies bind only to a single epitope on the virus surface, they may not provide adequate coverage, limiting their ability to block viral infection. In contrast, when multiple antibodies recognize and bind to different antigenic sites on the virus surface, they can more comprehensively occupy the surface, creating a multivalent blocking mechanism that enhances antiviral efficacy. Inspired by this, we prepared a “cocktail” of the seven VHHs with neutralizing activity and tested their combined effects. The results showed that this combination of single-domain antibodies performed better than individual antibodies alone, suggesting that in future applications, such as animal protection experiments, VHHs could be used in combination.

It is also important to note that to achieve high expression levels and obtain active VHHs, we optimized various expression systems and protein purification methods. Due to the limitations of adherent HEK293T cells for antibody expression, we selected the suspension-cultured FreeStyle 293-F cell line as the expression platform for VHH production. The FreeStyle 293-F cell line is widely used in biopharmaceuticals for its efficiency in antibody expression and the maturity of its associated technology [43]. In this study, after optimizing culture conditions, the viability of FreeStyle 293-F cells consistently exceeded 95%, and cell density increased significantly to 10^6^ cells/mL within 2–3 days of passage. High cell viability proved to be a key factor in the efficient expression of VHHs. Additionally, during the purification process, we encountered a challenge where the His tag was obscured due to protein folding, reducing purification efficiency. To address this, we treated the proteins with urea during purification to enhance solubility and improve efficiency. We found that desalting columns effectively remove urea and restore the biological activity of the antibodies, proving more efficient than traditional dialysis methods. Antibodies treated with desalting columns retained high activity in IFA and Western blot experiments. In conclusion, this study successfully established an efficient process for antibody expression and purification, providing a valuable reference for researchers working in related fields.

Research on using antibodies to treat human diseases is advanced, particularly in fields like cancer therapy and gene therapy, where monoclonal antibodies serve as carriers for targeted drugs, improving treatment precision and effectiveness. However, related research in veterinary medicine remains in early stages. In particular, reports on the development of neutralizing antibodies against PEDV are quite limited [44,45,46]. For instance, Shi Wenshu and colleagues developed a monoclonal antibody against the PEDV S protein (PEDV mAb-2) using fusion hybridoma technology, which was able to neutralize strains CV777 (G1 type), PEDV-SDSX16, and PEDV-Aj1102 (G2 type) [29]. Similarly, Zhang Fanqing et al. constructed a porcine phage-scFv library targeting key pathogens responsible for porcine diarrhea, screening out three scFvs against PEDV and confirming their antiviral roles during PEDV’s binding to cells. In vivo antibody prevention experiments also confirmed that piglets treated with oral scFvs could largely prevent PEDV infection [28]. In this study, we employed a novel approach by utilizing yeast two-hybrid technology to screen for nanobodies that specifically bind to the PEDV S protein and exhibit neutralizing efficiency, which has not been previously reported. The yeast library used in this study was constructed from an artificially synthesized nanobody library (Ruiyuan Biotechnology, Nanjing, China), which was derived from camelid antibody genes. Randomized sequences were introduced into the highly variable regions of the CDRs based on the characteristics of nanobodies. Evaluation revealed that the quantity and diversity of nanobodies generated from this synthetic library are comparable to those obtained through animal immunization. Through yeast surface display and high-throughput sequencing, the library was found to contain over 10^8^ full-length nanobody clones, which can be expressed and displayed on the yeast surface.

Neutralization experiments demonstrated that VHH1–4, VHH1–41, VHH1–89, VHH2–18, VHH2–28, VHH2–50, and VHH2–77 possess in vitro neutralizing activity, while VHH1–15 showed no neutralizing effect. In this study, the interactions between the selected PEDV S-VHHs and the S protein were analyzed using 3D structure modeling, molecular docking, and binding site prediction. The results indicated that all eight VHHs bind to the S protein, specifically targeting the S2 subunit, which partially explains their ability to bind specifically to PEDV. However, the reasons why VHH1–4, VHH1–41, VHH1–89, VHH2–18, VHH2–28, VHH2–50, and VHH2–77 exhibit neutralizing activity while VHH1–15 does not remain unclear. Furthermore, it is not yet fully understood how antibody binding to different regions of the S2 protein reduces viral infectivity in Vero E6 cells or what role these specific residues play during viral entry. Additional experimental validation is required to confirm these predictions and clarify the relationship between binding sites and viral neutralization capacity. According to the literature, most neutralizing epitopes identified so far are located in the S1 subunit, with relatively few antibodies binding to the S2 subunit. However, some studies suggest that antibodies targeting the S2 subunit can reduce viral infectivity in Vero E6 cells, as the S2 region is recognized as a critical functional domain during viral infection [44,45,46]. Our predictive data contribute to a deeper understanding of the antigenic structure of the PEDV S2 subunit, providing valuable insights for the future development of epitope-based vaccines.

To further develop PEDV nanobody therapeutics, future research should focus on the following. constructing stable FreeStyle 293-F cell lines expressing PEDV S-VHHs to increase antibody yield and simplify the preparation process; verifying the therapeutic efficacy of PEDV S-VHHs through animal antibody therapy experiments; and addressing the potential short half-life of nanobodies in animals by exploring the expression of nanobodies fused with the pig IgG1 Fc fragment to enhance their stability.

To further develop PEDV nanobody therapeutics, future research should focus on several key areas. One such area is constructing stable FreeStyle 293-F cell lines expressing PEDV S-VHHs to increase antibody yield and simplify the preparation process. Additionally, animal trials are essential to validate the therapeutic efficacy of PEDV S-VHHs. A proposed experimental design involves selecting age-matched piglets exhibiting comparable PEDV symptoms for the administration of oral antibodies. The efficacy of the PEDV S-VHHs treatment could be assessed using standardized clinical scoring criteria to evaluate the symptoms of the piglets. Furthermore, to address the potential issue of the short in vivo half-life of nanobodies, we could employ SOE-PCR to fuse the VHH gene with the Fc fragment of pig IgG1, creating a VHH-Fc fusion protein. This construct could be cloned into a eukaryotic expression vector, expressed, and purified to produce VHH-Fc antibodies, thus improving their half-life and stability in vivo.

## 5. Conclusions

In summary, eight PEDV S-VHHs specifically targeting the PEDV S protein were successfully identified using yeast two-hybrid technology. Their specificity in binding to the S protein and PEDV was validated through IFA and Western blotting. Neutralization assays further confirmed that seven of these PEDV S-VHHs possess neutralizing activity against PEDV, with a combination of these VHHs showing enhanced antiviral effects. Preliminary predictions of their binding sites on the PEDV S protein were also made. The PEDV S-VHHs identified in this study hold significant potential for further development of therapeutic antibodies, providing a strong theoretical foundation and research framework for the advancement of VHH-based treatments and preventive drugs for PEDV.

## Data Availability

Data are contained within the article and Supplementary Materials.

## Supplementary Materials

The following supporting information can be downloaded at: https://www.mdpi.com/article/10.3390/vetsci11110533/s1, Table S1: The amino acid sequence of the selected PEDV S-VHHs; Figure S1: The S gene sequence of the PEDV-JSXH-2021 strain; Figure S2: Western blotting was performed to detect the expression of PEDV S-VHHs in FreeStyle 293-F cells; Figure S3: IFA analysis of the binding of PEDV S-VHHs to PEDV; Table S2: Summary of neutralization assay data for different VHHs.
