# Supplementary material for: Neutralizing VHH Antibodies Targeting the Spike Protein of PEDV
Source: Vet Sci. 2024 Nov 1;11(11):533. doi: 10.3390/vetsci11110533 (PMC11598873; doi:10.3390/vetsci11110533)
Supplement: Supplementary file 1 [file vetsci-11-00533-s001.zip › vetsci-3238485-supplementary.pdf]

Table S1. The amino acid sequence of the selected PEDV S-VHHs.

The selected VHHs	The amino acid sequence of PEDV S-VHHs
VHH1–4	QVQLQESGGGLVQAGGSLRLSCAASGTIFCYGGMGWYRQAPGK<br>ERELVAGITMGTSTNYADSVKGRFTISRDNAKNTVYLQMNSLKPE<br>DTAVYYCAAAVMVAGPYQSWHGYWGQGTQVTVSS
VHH1–15	QVQLQESGGGLVQAGGSLRLSCAASGYISDAYYMGWYRQAPGK<br>EREFVATITHGTNTYYADSVKGRFTISRDNAKNTVYLQMNSLKPE<br>DTAVYYCAVLETRSYSFRYWGQGTQVTVSS
VHH1–41	QVQLQESGGGLVQAGGSLRLSCAASGSIFTFVRMGWYRQAPGK<br>ERELVAGIAIGSITYYADSVKGRFTISRDNAKNTVYLQMNSLKPED<br>TAVYYCAAQSVQKAETTSPFAYWGQGTQVTVSS
VHH1–89	QVQLQESGGGLVQAGGSLRLSCAASGYISDAYYMGWYRQAPGK<br>EREFVATITHGTNTYYADSVKGRFTISRDNAKNTVYLQMNSLKPE<br>DTAVYYCAVFLARIASFLPILLISLRYWGQGTQVTVSS
VHH2–18	QVQLQESGGGLVQAGGSLRLSCAASGYISDAYYMGWYRQAPGK<br>EREFVATITHGTNTYYADSVKGRFTISRDNAKNTVYLQMNSLKPE<br>DTAVYYCAASSLRKVLMTQLPTRLYYYWGQGTQVTVSS
VHH2–28	QVQLQESGGGLVQAGGSLRLSCAASGSISLHPQMGWYRQAPGK<br>ERELVAGIATGGNTNYADSVKGRFTISRDNAKNTVYLQMNSLKPE<br>DTAVYYCAVNEYHPRAMYALYIYWGQGTQVTVSS
VHH2–50	QVQLQESGGGLVQAGGSLRLSCAASGYISDAYYMGWYRQAPGK<br>EREFVATITHGTNTYYADSVKGRFTISRDNAKNTVYLQMNSLKPE<br>DTAVYYCAALFSIIVYYFSNSNLVFNYWGQGTQVTVSS
VHH2–77	QVQLQESGGGLVQAGGSLRLSCAASGYISDAYYMGWYRQAPGK<br>EREFVATITHGTNTYYADSVKGRFTISRDNAKNTVYLQMNSLKPE<br>DTAVYYCAVFSTISVNIYTFLYVIFFYWGQGTQVTVSS

Note: The red-marked sequence represents the CDR (complementarity determining region) sequence of PEDV S-VHHs.

ATGACGCCTTTAATTTACTTCTGGTTGTTCTTACCAGTACTTCTAACACTTAGCCTACCA
CAAGATGTCACTAGGTGCCAGTCTACTATTAACTTTAGGCGGTTCTTTTCAAAATTTAAT
GTTCAGGCACCTGCCGTCGTTGTTTTGGGTGGTTATCTACCTAGTATGAACTCTTCTAGC
TGGTACTGTGGCACAGGCATTGAAACTGATAGTGGCGTTCATGGTATTTTTCTCAGTTA
CATCGATTCTGGTCAGGGCTTTGAGATTGGCATTTCGCAAGAGCCGTTTGATCCTAGTG
GTTACCAGCTTTATTTACACAAGGCCACTAATGGTAACACTAGTGCTATTGCACGACTG
CGCATTTGCCAGTTTCCAGATAATAAAACATTGGGCCCTACTGTTAATGATGTTACAACA
GGTCGTAACTGCCTATTCAACAAAGCCATTCCAGCTTTGCAGGATGGAAAAAATATTGT
TGTCGGCATAACATGGGATAATGATCGTGTCACTGTTTTTGCTGACAAGATCTATCATTT
TTATATTAAAAATGATTGGTCCCGTGTTGCGACAAGATGTTACAATAAAAGAAGTTGTG

CCATGCAATATGTTTATACACCTACCTACTACATGCTTAATGTTACTAGTGCAGGTGAGG
ATGGCATTTACTATGAACCTTGTACAGCTAATTGCAGTGGTTACGCTGCCAATGTATTTG
CCACTGATTCCAATGGCCATATACCAGAAGGTTTTAGTTTTAATAATTGGTTTCTTTTGT
CCAATGACTCCACTTTGTTGCATGGTAAAGTGGTTTCAAACCAACCTTTGTTGGTCAAC
TGCCTTTGGGCCATTCCTAAGATTTATGGACTAGGCCAATTTTTCTCATTCAATCAAACG
ATGGATGGCGTTTGTAACGGAGCCGCTGCGCAGCGTGCCCCAGAGGCTCTGAGGTTTA
ATATTAATGACACTTTTGTCATTCTTGCTGAAGGCTCAATTGTACTTCATACTGCTTTAG
GAACAAATCTTTCTTTTGTTTGCAGTAATTCCTCAGATCCTCACAAAGCCATCTTTACCA
TACCTTTGGGTGTTACTGAAGTACCCTACTATTGCTTTCTTAAAGTGGATACTTACAAAT
CCACTGTTTATAAATTCTTGGCTGTTTTACCTCCTACTGTCAAGGAAATTGTCATCACCA
AGTACGGTGATGTTTATGTCAACGGGTTTGGCTATTTGCATCTCGGTTTGTTGGATGCTG
TCACAATTAATTTCACTGGTCATGGCACTGACGATGACGTTTCAGGTTTCTGGACCGTA
GCATCGACTAATTTTGTTGATGCACTCATCGAGGTTCAAGGAACTGCCATTCAGCGTAT
TCTTTATTGTGATGACCCTGTTAGCCAACTTAAGTGTTCTCAGGTTTCTTTTGACCTTGA
TGATGGTTTTTACCCTATTTCTTCTAGAAACCTTCTGAGTCATGAACAGCCAATTTCTTT
TGTTACTTTGCCATCATTCAATGATCATTCTTTTGTTAATATTACTGTCTCTGCGGCTTTT
GGTGGTCATAGTGGTGCCAACCTCATTGCATCTGACACTACTATCAATGGGTTTAGTTCT
TTCTGTGTTGACACTAGACAATTTACCATTACACTGTTTTATAACGTTACAAACAGTTAT
GGTTATGTGTCTAAGTCACAGGATAGTAATTGCCCTTTCACCTTGCAATCTGTTAATGAT
TACCTGTCTTTTAGCAAATTTTGTGTTTCAACCAGCCTTTTGGCTGGTGCTTGTACCATA
GATCTTTTTGGTTACCCTGAGTTCGGTAGTGGTGTTAAGTTTACGTCCCTTTATTTTCAA
TTCACAAAGGGTGAGTTGATTACTGGCACGCCTAAACCACTTCAAGGTGTCACGGACG
TTTCTTTTATGACTCTGGATGTGTGTACCAAGTATACTATCTATGGCTTTAAAGGTGAGG
GTATTATTACCCTTACAAATTCTAGCTTTTTGGCAGGTGTTTATTATACATCTGATTCTGG
ACAGTTGTTAGCCTTTAAGAATGTCACTAGTGGTGCTGTTTATTCTGTTACGCCATGTTC
TTTTTCAGAGCAGGCTGCATATGTTGATGATGATATAGTGGGTGTTATTTCTAGTTTGTCT
AACTCCACTTTTAACAATACCAGGGAGTTGCCTGGTTTCTTCTACCATTCTAATGATGGC
TCCAATTGTACAGAGCCTGTGTTGGTGTATAGTAACATAGGTGTCTGTAAATCTGGCAG
TATTGGCTATGTCCCACTTCAGGATGGCCAAGTCAAGATTGCACCCATGGTTACTGGGA
ATATTAGTATTCCCACCAACTTTAGTATGAGTATTAGAACAGAATATTTACAGCTTTACA
ACACGCCTGTTAGTGTTGATTGCGTTACATATGTTTGTAATGGTAACTCTCGTTGTAAAC
AATTACTCACCCAGTACACTGCAGCATGTAAGACCATAGAGTCAGCATTACAACTCAGC
GCTAGGCTTGAGTCTGTTGAAGTTAACTCTATGCTTACTATTTCTGAAGAGGCTCTACA
GTTAGCTACCATCAGTTCGTTTAATGGTGATGGATATAACTTTACTAATGTGCTGGGTGT
TTCCGTGTACGACCCTGCAAGTGGCAGGGTGGTACAAAAAGGGTCTTTTATTGAAGAC
CTGCTTTTTAATAAAGTGGTTACTAATGGCCTTGGTACTGTTGATGAAGACTATAAGCGC
TGTTCTAATGGTCGCTCTGTGGCAGATCTAGTCTGTGCGCAGTATTACTCTGGTGTCATG
GTACTACCTGGCGTTGTTGACGCTGAGAAGCTTCAAATGTATAGTGCGTCTCTCCTCGG
TGGTATGGCGCTAGGAGGTCTTACTACTGCAGCGGCATTGCCTTTTAGCAATGCTGTTC
AAGCGAGGCTCAATTATCTTGCTTTACAGACGGATGTTCTACAGCGCAACCAGCAATTG
CTTGCTGAGTCTTTTAACTCTGCTATTGGTAATATAACTTCAGCCTTTGAGAGTGTTAAA
GAGGCTATTAGTCAAACTTCCAATGGTTTGAACACTGTGGCTCATGCGCTTACTAAGGT
TCAAGAGGTTGTTAATTCGCAGGGTTCAGCTTTGACCCAACTTACCATACAGCTGCAAC
ACAACTTCCAAGCCATTTCTAGTTCTATTGATGACATTTACTCCCGACTGGACATTCTTT

CAGCCGATGTTCAGGTTGATCGTCTCATCACCGGCAGATTATCAGCACTTAATGCTTTT
GTTGCTCAAACCCTCACTAAGTATACTGAGGTTCAGGCTAGCAGGAAGCTAGCACAGC
AAAAGGTTAATGAGTGCGTCAAATCGCAATCTCAGCGTTATGGTTTTTGTGGTGGTGAT
GGCGAGCACATCTTCTCTCTGGTACAGGCCGCACCTCAGGGCCTGCTGTTCTTACATAC
AGTACTTGTACCGGGTGATTTTGTAAATGTTATTGCCATCGATGGCTTATGCGTTAATGG
TGATATTGCCTTGACTCTACGTGAGCCTGGCTTAGTCTTGTTTACGCATGAACTTCAAA
CTTATACTGCGACGGAATATTTTGTTTCATCGCGACGTATGTTTGAACCTAGAAAACCTA
CCGTTAGTGATTTTGTTCAAATTGAGAGTTGTGTGGGCACCTATGTCAATCTGACTAGC
GACCAACTACCAGATGTAATCCCAGATTACATCGATGTTAACAAAACACTTGATGAGAT
TCTAGCTTCTCTGCCCAATAGAATTGGTCCTAGTCTTCCCCTAGATGTTTTTAATGCCAC
TTATCTTAATCTCACTGGTGAAATTGCAGATTTAGAGCAGCGTTCAGAGTCTCTCCGTA
ATACTACAGAAGAGCTCCGAAGTCTCATATATAATATCAACAACACACTTGTTGACCTT
GAGTGGCTCAACCGAGTTGAGACATATATCAAGTGGCCGTGGTGGGTTTGGTTGATTAT
TTTTATTGTTCTCATCTTTGTTGTGTCATTATTAGTGTTCTGCTGCATTTCCACGGGTTGT
TGTGGATGCTGCGGTTGTTGCGGTGCTTGTTTTTCAGGTTGTTGTAGGGGTCCTAGACT
TCAACCTTACGAAGCTTTTGAAAAGGTCCACGTGCAGTGA

Figure S1. The S gene sequence of the PEDV-JSXH-2021 strain. The full-length S gene of the PEDV-JSXH-2021 strain was determined to be 4149 bp through sequencing. The S1 gene is 2199 bp, marked in blue, while the S2 gene is 1950 bp, presented in black.

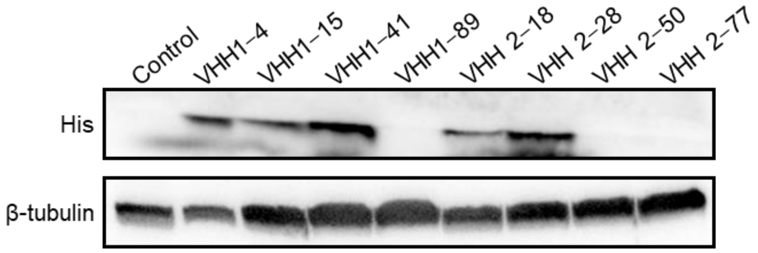

Figure S2. Western blotting was performed to detect the expression of PEDV S-VHHs in FreeStyle 293-F cells. Various VHH eukaryotic expression vectors were transfected into the cells, and cell pellets were collected 72 hours post-transfection. A mouse anti-His tag monoclonal antibody was used to detect the expression of VHH1–4, VHH1–15, VHH1–41, VHH1–89, VHH2–18, VHH2–28, VHH2–50, and VHH2–77, with the target protein being approximately 15 kDa. Cells transfected with the empty vector served as a control.

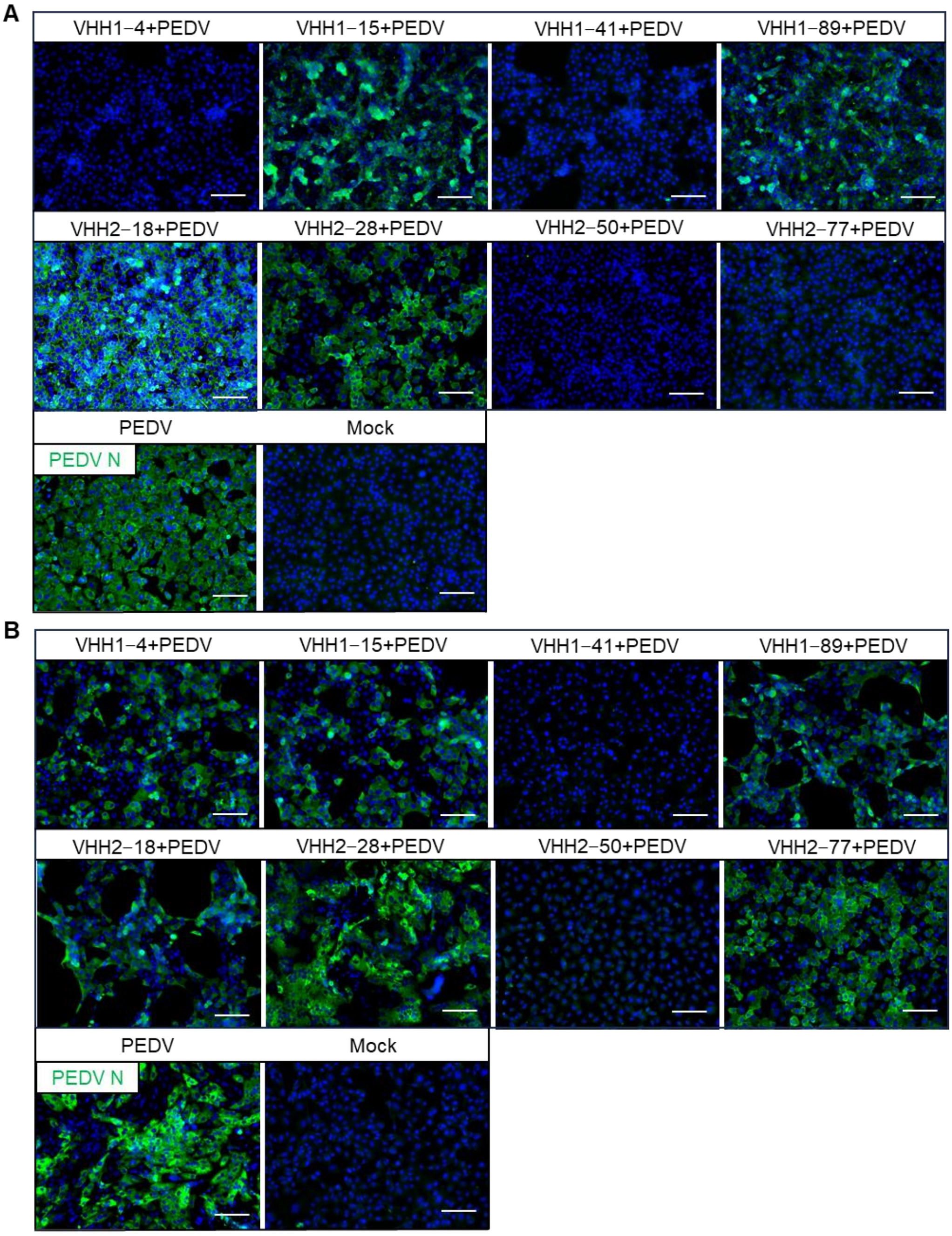

Figure S3. IFA analysis of the binding of PEDV S-VHHs to PEDV. (**A**) IFA detection of specific binding of PEDV S-VHHs to the CV777 strain. (**B**) IFA detection of specific binding of PEDV S-VHHs to the JXJA-2022 strain. Vero E6 cells infected with the CV777 or JXJA-2022 strain (MOI=0.01) for 48 hours were used for detection. PEDV S-VHHs were employed as the primary antibodies, with a mouse anti-His tag monoclonal antibody as the secondary antibody, and a goat anti-mouse IgG (H+L) conjugated with Alexa Fluor 488 as the tertiary antibody. Vero E6 cells not infected with CV777 and JXJA-2022 served as the mock control. The Vero E6 cells infected with the CV777 or JXJA-2022 strain were labeled with a mouse anti-PEDV N polyclonal antibody as the positive control. Scale bar = 100 μm.

Table S2. Summary of neutralization assay data for different VHHs.

VHH 1–4 concentration (ng/μL)	1st					2 st					3 st				
	Number of wells with CPE	Number of wells without CPE	Cumulative results		Neutralization test (%)	Number of wells with CPE	Number of wells without CPE	Cumulative results		Neutralization test (%)	Number of wells with CPE	Number of wells without CPE	Cumulative results		Neutralization test (%)
			Number of wells with CPE	Number of wells without CPE				Number of wells with CPE	Number of wells without CPE				Number of wells with CPE	Number of wells without CPE	
100	0	4	0	4	100	0	4	0	4	100	0	4	0	5	100
50	4	0	4	0	0	4	0	4	0	0	3	1	3	1	25
25	4	0	8	0	0	4	0	8	0	0	4	0	7	0	0
12.5	4	0	12	0	0	4	0	12	0	0	4	0	11	0	0
6.25	4	0	16	0	0	4	0	16	0	0	4	0	15	0	0
3.125	4	0	20	0	0	4	0	20	0	0	4	0	19	0	0

VHH 1–15 concentration (ng/μL)	1st					2 st					3 st				
	Number of wells with CPE	Number of wells without CPE	Cumulative results		Neutralization test (%)	Number of wells with CPE	Number of wells without CPE	Cumulative results		Neutralization test (%)	Number of wells with CPE	Number of wells without CPE	Cumulative results		Neutralization test (%)
			Number of wells with CPE	Number of wells without CPE				Number of wells with CPE	Number of wells without CPE				Number of wells with CPE	Number of wells without CPE	
100	4	0	4	0	0	4	0	4	0	0	4	0	4	0	0
50	4	0	8	0	0	4	0	8	0	0	4	0	8	0	0
25	4	0	12	0	0	4	0	12	0	0	4	0	12	0	0
12.5	4	0	16	0	0	4	0	16	0	0	4	0	16	0	0
6.25	4	0	20	0	0	4	0	20	0	0	4	0	20	0	0
3.125	4	0	24	0	0	4	0	24	0	0	4	0	24	0	0

VHH 1–41 concentration (ng/μL)	1st					2 st					3 st				
	Number of wells with CPE	Number of wells without CPE	Cumulative results		Neutralization test (%)	Number of wells with CPE	Number of wells without CPE	Cumulative results		Neutralization test (%)	Number of wells with CPE	Number of wells without CPE	Cumulative results		Neutralization test (%)
			Number of wells with CPE	Number of wells without CPE				Number of wells with CPE	Number of wells without CPE				Number of wells with CPE	Number of wells without CPE	
100	0	4	0	14	100	0	4	0	13	100	0	4	0	13	100
50	0	4	0	10	100	0	4	0	9	100	0	4	0	9	100
25	0	4	0	6	100	0	4	0	5	100	0	4	0	5	100
12.5	2	2	2	2	50	3	1	3	1	25	3	1	3	1	25
6.25	4	0	6	0	0	4	0	7	0	0	4	0	7	0	0
3.125	4	0	10	0	0	4	0	11	0	0	4	0	11	0	0

VHH 1–89 concentration (ng/μL)	1st					2 st					3 st				
			Cumulative results					Cumulative results					Cumulative results		
	Number of wells with CPE	Number of wells without CPE	Number of wells with CPE	Number of wells without CPE	Neutralization test (%)	Number of wells with CPE	Number of wells without CPE	Number of wells with CPE	Number of wells without CPE	Neutralization test (%)	Number of wells with CPE	Number of wells without CPE	Number of wells with CPE	Number of wells without CPE	Neutralization test (%)
100	0	4	0	18	100	0	4	0	17	100	0	4	0	16	100
50	0	4	0	14	100	0	4	0	13	100	0	4	0	12	100
25	0	4	0	10	100	0	4	0	9	100	0	4	0	8	100
12.5	0	4	0	6	100	0	4	0	5	100	0	4	0	4	100
6.25	2	2	2	2	50	3	1	3	1	25	4	0	4	0	0
3.125	4	0	6	0	0	4	0	7	0	0	4	0	8	0	0

VHH 2–18 concentration (ng/μL)	1st					2 st					3 st				
			Cumulative results					Cumulative results					Cumulative results		
	Number of wells with CPE	Number of wells without CPE	Number of wells with CPE	Number of wells without CPE	Neutralization test (%)	Number of wells with CPE	Number of wells without CPE	Number of wells with CPE	Number of wells without CPE	Neutralization test (%)	Number of wells with CPE	Number of wells without CPE	Number of wells with CPE	Number of wells without CPE	Neutralization test (%)
100	0	4	0	10	100	0	4	0	10	100	0	4	0	12	100
50	0	4	0	6	100	0	4	0	6	100	0	4	0	8	100
25	3	1	3	2	40	2	2	2	2	50	0	4	0	4	100
12.5	3	1	6	1	14.29	4	0	6	0	0	4	0	4	0	0
6.25	4	0	10	0	0	4	0	10	0	0	4	0	8	0	0
3.125	4	0	14	0	0	4	0	14	0	0	4	0	12	0	0

VHH 2–28 concentration (ng/μL)	1st					2 st					3 st				
			Cumulative results					Cumulative results					Cumulative results		
	Number of wells with CPE	Number of wells without CPE	Number of wells with CPE	Number of wells without CPE	Neutralization test (%)	Number of wells with CPE	Number of wells without CPE	Number of wells with CPE	Number of wells without CPE	Neutralization test (%)	Number of wells with CPE	Number of wells without CPE	Number of wells with CPE	Number of wells without CPE	Neutralization test (%)
100	0	4	0	9	100	0	4	0	10	100	0	4	0	8	100
50	0	4	0	5	100	0	4	0	6	100	0	4	0	4	100
25	3	1	3	1	25	2	2	2	2	50	4	0	4	0	0
12.5	4	0	7	0	0	4	0	6	0	0	4	0	8	0	0
6.25	4	0	11	0	0	4	0	10	0	0	4	0	12	0	0
3.125	4	0	15	0	0	4	0	14	0	0	4	0	16	0	0

VHH 2–50 concentration (ng/μL)	1st					2 st					3 st				
	Number of wells with CPE	Number of wells without CPE	Cumulative results		Neutralization test （%）	Number of wells with CPE	Number of wells without CPE	Cumulative results		Neutralization test （%）	Number of wells with CPE	Number of wells without CPE	Cumulative results		Neutralization test （%）
			Number of wells with CPE	Number of wells without CPE				Number of wells with CPE	Number of wells without CPE				Number of wells with CPE	Number of wells without CPE	
100	0	4	0	15	100	0	4	0	12	100	0	4	0	13	100
50	0	4	0	11	100	0	4	0	8	100	0	4	0	9	100
25	0	4	0	7	100	0	4	0	4	100	0	4	0	5	100
12.5	2	2	2	3	60	4	0	4	0	0	3	1	3	1	25
6.25	3	1	5	1	16.67	4	0	8	0	0	4	0	7	0	0
3.125	4	0	24	0	0	4	0	24	0	0	4	0	24	0	0

VHH 2–77 concentration (ng/μL)	1st					2 st					3 st				
	Number of wells with CPE	Number of wells without CPE	Cumulative results		Neutralization test （%）	Number of wells with CPE	Number of wells without CPE	Cumulative results		Neutralization test （%）	Number of wells with CPE	Number of wells without CPE	Cumulative results		Neutralization test （%）
			Number of wells with CPE	Number of wells without CPE				Number of wells with CPE	Number of wells without CPE				Number of wells with CPE	Number of wells without CPE	
100	0	4	0	17	100	0	4	0	16	100	0	4	0	17	100
50	0	4	0	13	100	0	4	0	12	100	0	4	0	13	100
25	0	4	0	9	100	0	4	0	8	100	0	4	0	9	100
12.5	0	4	0	5	100	0	4	0	4	100	0	4	0	5	100
6.25	3	1	3	1	25	4	0	4	0	0	3	1	3	1	25
3.125	4	0	7	0	0	4	0	8	0	0	4	0	7	0	0

VHHs concentration (ng/μL)	1st					2 st					3 st				
	Number of wells with CPE	Number of wells without CPE	Cumulative results		Neutralization test （%）	Number of wells with CPE	Number of wells without CPE	Cumulative results		Neutralization test （%）	Number of wells with CPE	Number of wells without CPE	Cumulative results		Neutralization test （%）
			Number of wells with CPE	Number of wells without CPE				Number of wells with CPE	Number of wells without CPE				Number of wells with CPE	Number of wells without CPE	
100	0	4	0	21	100	0	4	0	19	100	0	4	0	21	100
50	0	4	0	17	100	0	4	0	15	100	0	4	0	17	100
25	0	4	0	13	100	0	4	0	11	100	0	4	0	13	100
12.5	0	4	0	9	100	0	4	0	7	100	0	4	0	9	100
6.25	0	4	0	5	100	1	3	1	3	75	0	4	0	5	100
3.125	3	1	3	1	25	4	0	5	0	0	3	1	3	1	25

PEDV N antibody dilution	1st					2 st					3 st				
	Number of wells with CPE	Number of wells without CPE	Cumulative results		Neutralization test （%）	Number of wells with CPE	Number of wells without CPE	Cumulative results		Neutralization test （%）	Number of wells with CPE	Number of wells without CPE	Cumulative results		Neutralization test （%）
			Number of wells with CPE	Number of wells without CPE				Number of wells with CPE	Number of wells without CPE				Number of wells with CPE	Number of wells without CPE	
1:2	4	0	4	0	0	4	0	4	0	0	4	0	4	0	0
1:4	4	0	8	0	0	4	0	8	0	0	4	0	8	0	0
1:8	4	0	12	0	0	4	0	12	0	0	4	0	12	0	0
1:16	4	0	16	0	0	4	0	16	0	0	4	0	16	0	0
1:32	4	0	20	0	0	4	0	20	0	0	4	0	20	0	0
1:64	4	0	24	0	0	4	0	24	0	0	4	0	24	0	0
