# Supplementary figures and images for: Neutralizing VHH Antibodies Targeting the Spike Protein of PEDV
Source: Vet Sci. 2024 Nov 1;11(11):533. doi: 10.3390/vetsci11110533 (PMC11598873; doi:10.3390/vetsci11110533)

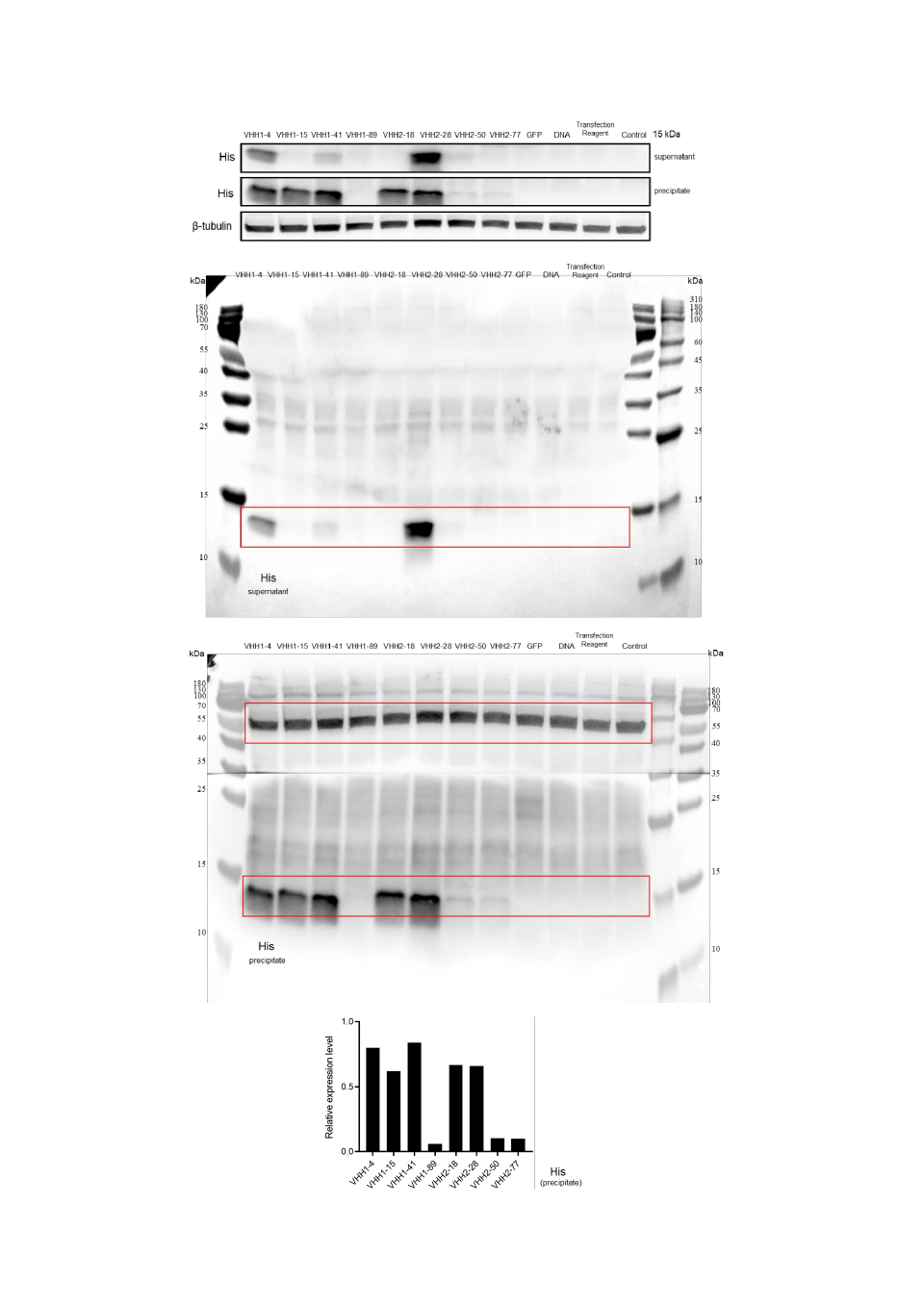

Supplement: Supplementary file 1 [file vetsci-11-00533-s001.zip › Figure 2 supply.png]

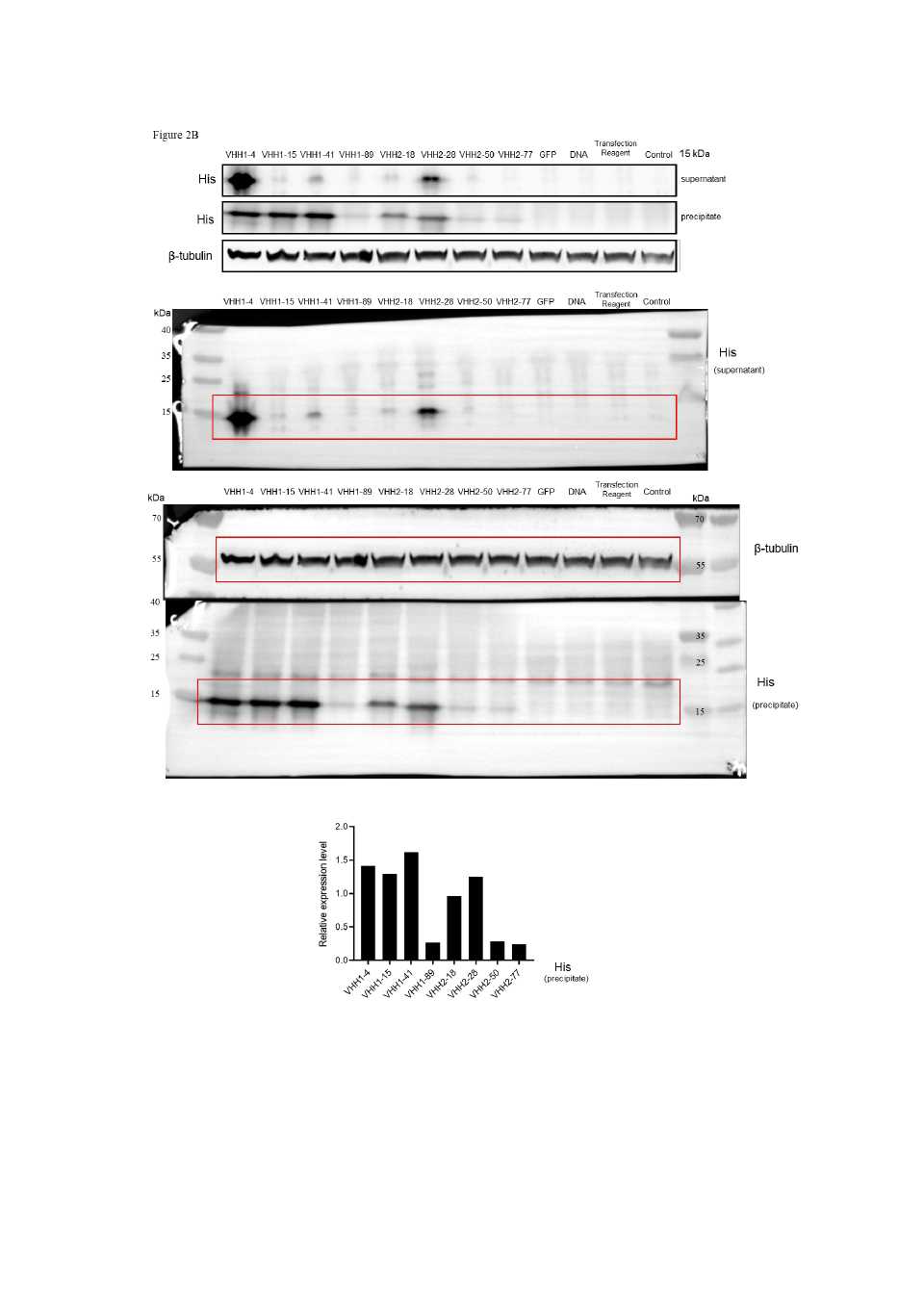

Supplement: Supplementary file 1 [file vetsci-11-00533-s001.zip › WB for Figure 2B.png]

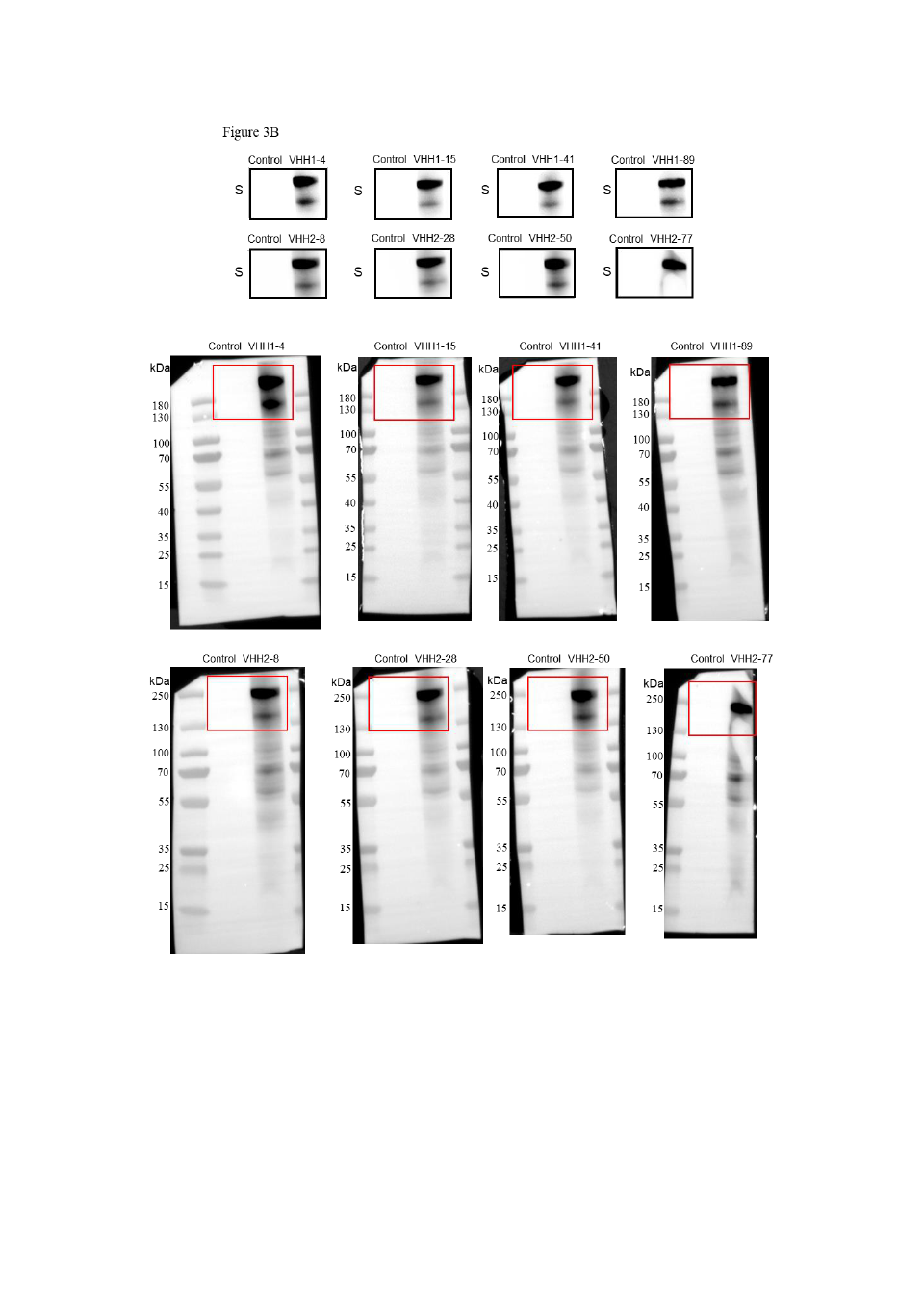

Supplement: Supplementary file 1 [file vetsci-11-00533-s001.zip › WB for figure 3B.png]

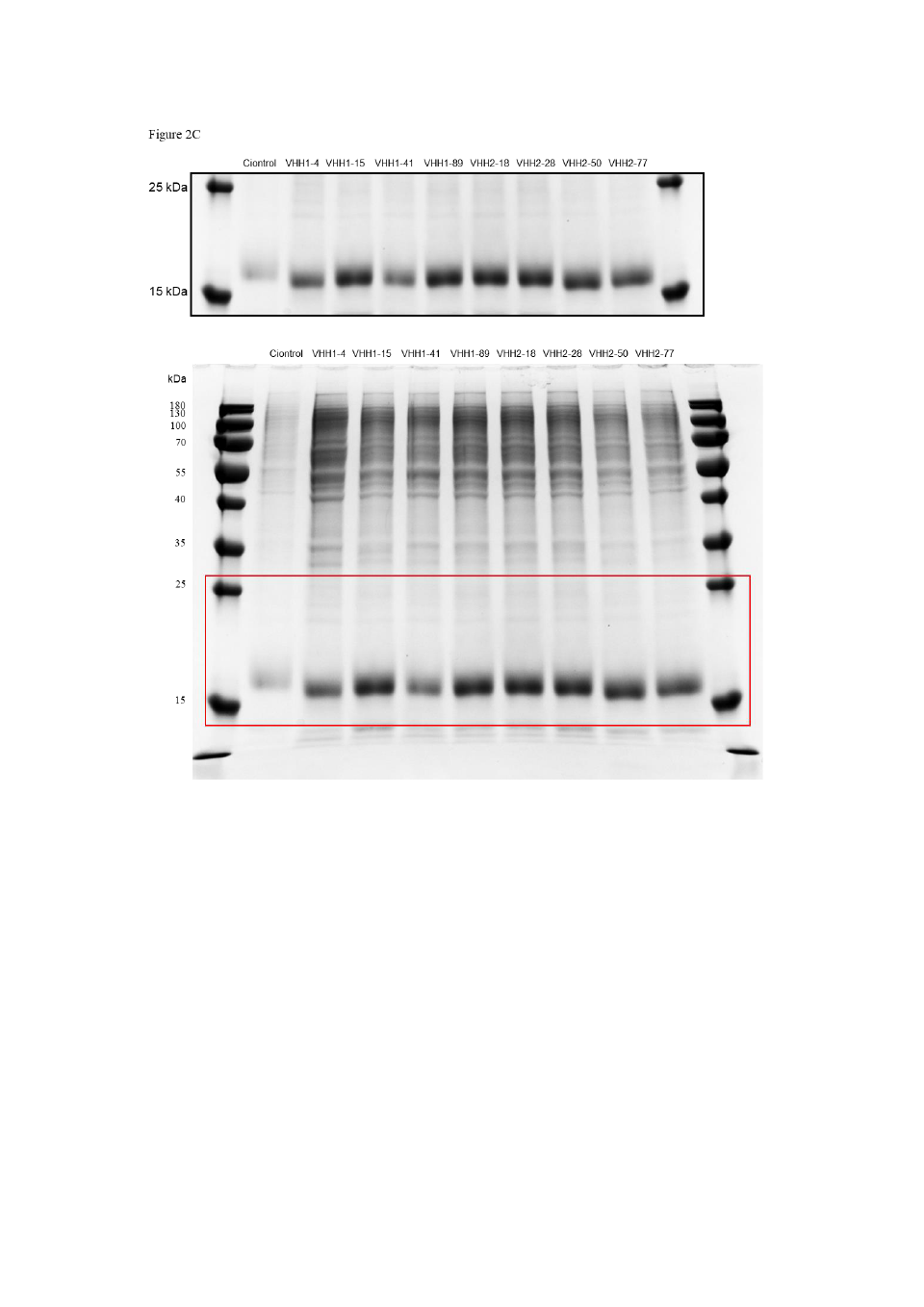

Supplement: Supplementary file 1 [file vetsci-11-00533-s001.zip › WB for Figure2c.png]
